# Integration of modeling and simulation into hospital-based decision support systems guiding pediatric pharmacotherapy

**DOI:** 10.1186/1472-6947-8-6

**Published:** 2008-01-28

**Authors:** Jeffrey S Barrett, John T Mondick, Mahesh Narayan, Kalpana Vijayakumar, Sundararajan Vijayakumar

**Affiliations:** 1 Department of Pediatrics, Division of Clinical Pharmacology and Therapeutics, The Children's Hospital of Philadelphia, USA; 2 INTEK Partners, Inc., 676 Route 202-206 North, Bridgewater, NJ 08807, USA

## Abstract

**Background:**

Decision analysis in hospital-based settings is becoming more common place. The application of modeling and simulation approaches has likewise become more prevalent in order to support decision analytics. With respect to clinical decision making at the level of the patient, modeling and simulation approaches have been used to study and forecast treatment options, examine and rate caregiver performance and assign resources (staffing, beds, patient throughput). There us a great need to facilitate pharmacotherapeutic decision making in pediatrics given the often limited data available to guide dosing and manage patient response. We have employed nonlinear mixed effect models and Bayesian forecasting algorithms coupled with data summary and visualization tools to create drug-specific decision support systems that utilize individualized patient data from our electronic medical records systems.

**Methods:**

Pharmacokinetic and pharmacodynamic nonlinear mixed-effect models of specific drugs are generated based on historical data in relevant pediatric populations or from adults when no pediatric data is available. These models are re-executed with individual patient data allowing for patient-specific guidance via a Bayesian forecasting approach. The models are called and executed in an interactive manner through our web-based dashboard environment which interfaces to the hospital's electronic medical records system.

**Results:**

The methotrexate dashboard utilizes a two-compartment, population-based, PK mixed-effect model to project patient response to specific dosing events. Projected plasma concentrations are viewable against protocol-specific nomograms to provide dosing guidance for potential rescue therapy with leucovorin. These data are also viewable against common biomarkers used to assess patient safety (e.g., vital signs and plasma creatinine levels). As additional data become available via therapeutic drug monitoring, the model is re-executed and projections are revised.

**Conclusion:**

The management of pediatric pharmacotherapy can be greatly enhanced via the immediate feedback provided by decision analytics which incorporate the current, best-available knowledge pertaining to dose-exposure and exposure-response relationships, especially for narrow therapeutic agents that are difficult to manage.

## Background

Decision making in a hospital environment occurs at multiple levels of the organization and in a variety of departmental settings. Likewise, numerous stakeholders including hospital administration, staffing planners, facilities management, pharmacy administration, caregivers and physicians as well as healthcare providers are reliant on decision support systems (DSS) to facilitate decision making in their specific areas. Ultimately, the outcome, at least in theory, should be better decisions yielding more efficient provision of services and optimal (most appropriate and cost-effective) patient care. Informing today's decision makers is a cadre of tools and decision analytics. Historically, hospital environments have not been the hallmark of innovation in decision analytics and the discrepancy between the hospital environment and other industries has received a great deal of attention recently. In late 2005, the National Academies of Engineering and Institute of Medicine issued a joint report that cited the urgency and importance of bringing contemporary System Engineering techniques to healthcare. Annual gross waste of a staggering 30–40% of every dollar spent in healthcare and continued medical errors that cause nearly 100,000 patient deaths and serious injuries yearly were only two of the more serious problems covered in the report [1,2]. This situation is changing in a dramatic manner as information technology, engineering, clinical and informatics scientists collaborate on analytic approaches that address decision requirements of the current inpatient environment.

The backbone of many decision support systems typically consist of transactional and/or relational databases and models which describe, predict and/or simulate response to varied inputs. Table 1 illustrates the diversity in modeling and simulation application in the hospital setting [3-21]. The examples are representative as this is certainly not an exhaustive account. Along with the varied applications are diverse physical and stochastic models and analytics. The choice of approach is dependent upon the objectives of the application of course but there exists a great deal of overlap between modeling approaches and procedures yielding the possibility of multiple solutions to decision making questions. Our purpose is not to review such approaches (although such a review is needed) but rather to focus on the use of nonlinear mixed effect modeling to guide decisions regarding the use and management of drugs to treat patients – pharmacotherapy.

**Table 1:** Diversity in modeling and simulation applications in the hospital setting

**M&S Fields**	**Application**	**Approach**
Treatment Outcomes	• Medical folder management system – physician clinical decision making [3] • Cancer pharmacotherapy multi-drug decision support [4] • Predictive model to predict Clostridium difficile infection (diahhrea) outbreaks [5] • Hospital-wide surveillance for nosocomial infection to assess patient risk [6] • Methicillin-resistant Staphylococcus aureus transmission among hospitalized patients – risk factors and prediction [7]	• DSS interfaced to EMRS • KITT model and decision tree • Reversible jump MCMC model • Logistic regression model • Monte Carlo simulation

Healthcare Costs	• Health care costs of geriatric inpatients [8] • Hospital-acquired infection costs [9] • Costs and outcomes of cardiovascular surgery [10]	• Bayesian Network Theory/Model • Monte Carlo simulation model • Systems dynamic model STELLA

Patient Flow/Occupancy	• Patient flow in a pediatric emergency department [11] • Critical care planning capacity [12] • Healthcare facility patient flow [13] • Hospital patient flow [13]	• Discrete event simulation • CART analysis • Queuing network system • Clustering

Hospital Operations	• Hospital operations for emergency response [15] • Length of stay in the ICU [16] • Directly observed therapy in newly diagnosed HIV infection [17] • System-level investigation of emergency department (ED) operations [18] • Healthcare quality improvement via simulation [19] • Optimum operating room staffing needs for trauma centers [20] • ICU duration/length of stay analysis [21]	• Transient modeling regression approach • Linear regression • Probabilistic Markov Model • Discrete event simulation (EDSIM) • Multivariate simulation models • Queuing and simulation methods • Class probability tree

Pharmacotherapy is generally concerned with the safe and effective management of drug administration. It implies an understanding of drug pharmacokinetics (PK) and pharmacodynamics (PD) so that individual dosing guidance, when necessary, can be provided to optimize patient response within their individual therapeutic window. Pediatric pharmacotherapy can be challenging due to developmental changes that may alter drug kinetics, pathophysiologic differences that may alter pharmacodynamics, disease etiologies that may be different from adults, and other factors that may result in great variation in safety and efficacy outcomes. The situation becomes more convoluted when one considers children and the paucity of well-controlled pediatric clinical trials. This situation, despite the efforts of the Food and Drug Administration and the US Congress, is not likely to improve substantially due to the economic reality of the pediatric market.

**Figure 1:**
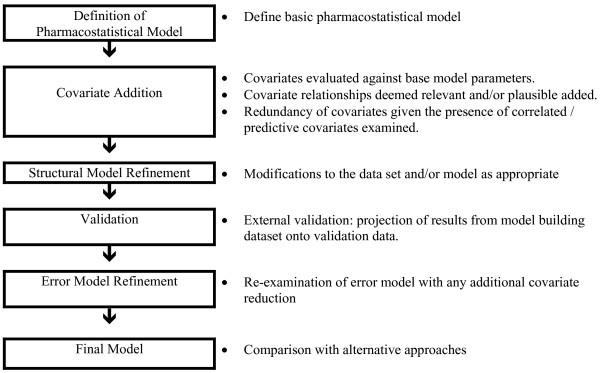
Typical progression of pharmacometric model development commonly used to support pharmacotherapeutic decision support systems.

Population pharmacokinetics is an approach to explain sources of variation in various pharmacokinetic processes across individual patient populations [22,23]. The nature of the sources of variation varies with drug and the underlying pharmacokinetic (structural) model can be simple or complex. Variation is also relevant to the population being studied and pediatrics represents a particularly dynamic setting given the many developmental changes which occur as children develop [24]. Nonlinear mixed effects models involve both fixed effects and random effects. Model building for nonlinear mixed effects is the process of determining the characteristics of both the fixed and the random effects in order to produce a predictive, generalizable and parsimonious model. Procedures based on information criterion statistics for comparing different structures of the random effects component are generally suitable to achieve a model which is both adequate to explain the sample data and, upon validation against an external data set, generalizable to the broader population (see Figure 1). Historically, therapeutic drug monitoring (TDM) has been used to monitor/guide pharmacotherapy particularly for agents with a narrow therapeutic index. Often, the monitored drug levels are judged against historical "norms" and not necessarily viewed in the context of specific factors which may suggest a different scale by which they should be judged. Also, time dependencies are seldom considered in these static devices. A model-based approach on the contrary has the ability to both account for demonstrated relevant patient factors (covariate model) and/or group (disease status) factors. While some attempts have been made to interface TDM data to therapeutic drug models, these have been developed for standalone applications and not with the physician caregiver in mind [25,26].

We have begun to interface our electronic medical record system with decision support analytics (drug dashboards) that summarize individual patient records and assemble the most relevant clinical data associated with drug therapy. Data visualization tools summarize patient profiles of lab values, vital signs, and associated biomarkers into tables and plots based on user-defined requirements. More importantly, this data populates models that predict future events as drug therapy continues. A prototype methotrexate dashboard is demonstrated. A population-based pharmacokinetic model (nonlinear mixed effect model) is used to simulate individual patient MTX drug concentrations based on that patient's current dosing regimen and compares expected exposures with nomograms that predict toxicity. The compilation of additional dashboards is planned for the construction of a larger pediatric knowledgebase (PKB) currently under construction. Our objective is to define the general approach of pharmacostatistical model building while demonstrating how such models can be interfaced to electronic medical record data front-ended by a web-based decision support system.

## Methods

### Population Pharmacokinetics

Population pharmacokinetics is the study of the sources and correlates of variability in drug concentrations among individuals who represent the target patient population receiving clinically relevant doses of a drug of interest. The goal of population pharmacokinetic analysis to identify pathophysiologic factors that cause changes in the dose-concentration relationship and the extent of these changes so that, if such changes are associated with clinically significant shifts in the therapeutic index, dosage can be appropriately modified. Population pharmacokinetic models adhere into a hierarchical structure. At the initial stage, the relationship between concentration and time (pharmacokinetics) is modeled for an individual patient. At the second stage, pharmacokinetic parameters that define each of the individual patients' drug concentration profiles are assigned some distributional form, after accounting for relevant covariate information. A primary aim of a population analysis is to determine covariates that are important predictors of pharmacokinetic parameters. A Bayesian model then requires a third stage in which prior distributions are specified for the parameters defining the second-stage distributional form and the intra-individual variance parameters. Such a Bayesian model defines and estimates the variability that is observed both in individual concentrations and between different individuals' pharmacokinetic parameters. This model framework makes it is possible to determine appropriate patient-specific dosage regimens that ensure the attainment of desirable drug concentrations.

The estimation method most commonly used in population pharmacokinetics and nonlinear mixed effect modeling in general is based on a maximum likelihood approach. Maximum Likelihood (ML) is an alternative to the least squares objective function; it seeks to maximize the *likelihood *or *log*-likelihood function (or to minimize the negative log-likelihood function). In general terms, the likelihood function is defined as:

$$\text{L}=\text{F(Y, Model)}=\Pi_{\text{i=1}}^{\text{n}}{\text{ \{p [y}}_{\text{i}}{\text{, Model Parameters (x}}_{\text{i}}\text{)]\}}$$

The probability (now called *L*, the *likelihood*) is predicted in the sample data, given the respective regression model. Provided that all observations are independent of each other, this likelihood is the geometric sum (Π, across *i *= 1 to *n *cases) of probabilities for each individual observation (*i*) to occur, given the respective model and parameters (*θ*'s) for the *x *values. As it is customary to express this function as a natural logarithm, the geometric sum becomes a regular arithmetic sum (Σ, across *i *= 1 to *n *cases). The larger the likelihood of the model, the larger is the probability of the dependent variable values to occur in the sample and the better is the fit of the model to the data. If all assumptions for standard multiple regression are met, then the standard least squares estimation method will yield results identical to the maximum likelihood method. If the assumption of equal error variances across the range of the *x *variable(s) is violated, then the weighted least squares method will yield maximum likelihood estimates.

The typical structural model is chosen from one of several compartmental models which incorporate the route of administration as a fixed input into the model with certain assumptions (i.e., linear or zero order input). Compartmental models are, for the most part, empirical even though they may incorporate some mechanistic assumptions so they appear more realistic. Numerically, they are generally easier to handle as opposed to mechanistic models. Complex mechanistic and/or highly parameterized structural models can be accommodated as well of course. The prediction engine discussed herein is not limited by the nature of the model definition.

The framework for the mixed effect modeling approach to population pharmacokinetic analysis can be defined as follows: for i = 1, ... n individuals in a population of interest, let x_ij_, j = 1, ... n_j _represent the design points on which the y_ij _responses are observed. In the pharmacokinetic (PK) setting, x_ij _are typically the sampling time points and y_ij _are the observed concentrations in the biologic matrix of interest (usually plasma or blood). Hence, the PK response can be described by

y_ij _= *f*(*θ*_i_, x_ij_) + *ε*_ij_

where the function *f *denotes the structural model; *θ*_i _is the p × 1 parameter vector for the i^th ^individual and *ε*_ij _are the independently and identically distributed (*i.i.d*.) error terms assumed to be normal random variables with a zero mean and a variance (*σ*_e_^2^) which may depend on the mean concentration. The *ε*_ij_'s account for the intraindividual variability and may incorporate model misspecification or other unresolved (or incorrect) error partitioning. In most population pharmacokinetic software the structural model is chosen from a library of compartmental models, expressed as a closed form system of equations or defined via differential equations. The probability density function which accounts for the within-individual variability only as

p(y_ij _| *θ*_i_, x_ij_)

The intra-individual variation about the ith individual is defined when the distribution *ε*_i _of is specified. The second stage model defines the between-individual variability in the parameters as follows

*θ*_i _= *θ *+ *η*_i_

where *θ *is the mean parameter vector for the population and *η*_i _are the individual deviations assumed to be *i.i.d*. and normal with zero mean vector and covariance matrix Ω. The expression of *θ*_i _shown (additive) is one of numerous ways that individual *θ*'s can be defined. In addition, the population *θ *can be expressed as a function of covariates (β_i_). The covariate matrix Ω captures both the variance and covariance among the *η*'s. The density of the second stage model can then be defined as

p(*θ*_i _| *θ*, Ω, β_i_)

where β_i _represents the individual patient covariate data (i.e., age, sex, race, etc.). The third stage of the mixed effect model approach would represent a Bayesian representation in which the model would contain the prior distributions of the population parameters as mentioned previously.

### Prediction Models for Toxicity and Adverse Events

PK/PD models can be developed to explore the relationship between drug exposure and observed toxicity as well. Endpoints can be expressed as a dichotomous categorical variable representing the occurrence of an adverse effect (AE, e.g., nausea, vomiting) or drug reaction (1 = yes, 0 = no). The population PD data are viewed as a probabilistic outcome and analyzed using a logistic regression model [27]. The probability of an event for individual *i *at sampling time *j *is given by *p*_*ij*_. The ratio of the probability of that event occurring vs. the probability that it does not occur is given by the Odds Ratio; the log of the Odds Ratio is known as the logit function (*λ*_*ij*_):

$$\begin{array}{ll} p_{ij}=exp^{\lambda_{ij}}/(1+exp^{\lambda_{ij}})\; & Probability\\ \frac{p_{ij}}{\left(1-p_{ij}\right)}=exp^{\lambda_{ij}}\; & OddsRatio\\ log\left[\frac{p_{ij}}{\left(1-p_{ij}\right)}\right]=\lambda_{ij} & \end{array}$$

Independent variables and covariates (*x*_*ij*_) will be incorporated into the model via the logit function, with population typical population (*θ*) and individual random effect (*η*_*i*_) parameters to be estimated:

*λ*_*ij *_= *f*(*x*_*ij*_, *θ*, *η*_*i*_)

Covariate effects, and random effects can modulate the predicted probability in a positive or negative direction, with the probability constrained between the values of 0 and 1.

In these analyses it is expected that each individual will contribute only one observation for each outcome endpoint. Thus the individual random effects (*η*_*i*_) will be fixed at a value of zero and a naïve-pooled data analysis will be conducted. This approach has also been suggested as a check for nonlinear mixed-effects models of dichotomous outcome data[28]. The predicted likelihood (*l*_*ij *_for individual *i *and sampling time *j*) of the data (*y*_*ij*_), given the model and parameters will be described by a binomial probability density function:

$$l_{ij}={\left[\exp^{\lambda_{ij}}/(1+\exp^{\lambda_{ij}})\right]}^{y_{ij}}\cdot {\left[1/(1+\exp^{\lambda_{ij}})\right]}^{1-y_{ij}}$$

The likelihood for the entire population PD data set is simply the product of likelihoods across all individuals and data points. Diagnostic plots and the minimum value of the objective function are used to guide model building and assess goodness-of-fit.

### Methotrexate (MTX) Model

The administration of methotrexate (MTX) to children with cancer was chosen as our initial setting to develop the first drug dashboard prototype. The difficulty in effectively administering high-dose MTX to oncology patients lie in balancing efficacy and safety. Increased MTX exposure has been shown to be predictive of greater efficacy [29-31], while increased MTX concentrations and prolonged exposure time have also been linked to toxicity [32]. Due to the high inter- and intra-patient variability in methotrexate pharmacokinetics, monitoring of methotrexate plasma concentrations in individual patients has become a standard procedure in order to identify patients at risk of toxicity. Typically, patient plasma concentrations are monitored starting at 24 hours post infusion until MTX plasma concentrations fall below 0.1 to 1 μM [33-37], with adjunct rescue therapy implemented as needed.

The occurrence of methotrexate-induced renal toxicity further complicates chemotherapy administration. Although methotrexate-induced nephrotoxicity is a relatively rare occurrence, it is none-the-less a life threatening complication of methotrexate therapy [38]. Since methotrexate is mainly cleared from systemic circulation via glomerular filtration and renal secretion, delayed drug elimination is a product of this nephrotoxicity. This results in prolonged drug exposure and elevated plasma concentrations. As a result of this increased exposure, severe adverse events such as myelosuppression, mucositis, and hepatitis become more prevalent and severe.

Numerous studies have been conducted to examine the feasibility and reliability of applying Bayesian forecasting approaches to predicting MTX pharmacokinetics. The goals of these studies have been to predict MTX concentrations at later times or the time that MTX concentrations fall below a threshold value [39-41], MTX dose adjustment [42,43], or providing guidance for rescue administration in the case of elevated MTX concentrations for prolonged time periods [44]. The Bayesian prediction models developed thus far have concentrated on those patients with normal renal function, and are not applicable in the case of severe renal dysfunction secondary to high-dose MTX administration.

We have developed a population pharmacokinetic model to implement as a Bayesian predictor of MTX concentrations in patients with normal renal function and MTX-induced renal dysfunction. Plasma concentrations from patients with normal renal function and patients with MTX-induced renal dysfunction were obtained from standard clinical monitoring. The model was constructed from methotrexate dosing histories and monitored drug concentrations in 240 patients. The original dataset contained 2176 observations covering a range of one to 56 observations per patient (an average of 9 observations per patient). The age range was from 1 to 80 years with a weight range of 6.6 to 157 kg. The gender distribution was approximately 48% male (52% female). Hence, our underlying patient diversity allowed us to include and consider relevant size and demographic dependencies. The model was developed using NONMEM version VI[45].

Methotrexate disposition is described by a two-compartment model with first-order elimination. Although MTX clearance changes over time in patients with renal dysfunction, clearance is approximated with a simple model defined by two different clearance distributions for the two populations. Inter-subject variability in PK parameters was expressed using an exponential error model:

$$P_{i}=\hat{P}\exp(\eta^{Pi})$$

where:

*P*_*i *_is the estimated parameter value for individual *i*

$\hat{P}$ is the typical population value (geometric mean) of the parameter

*η*^*Pi *^are individual-specific interindividual random effects for individual *i *and parameter *P *and are assumed to be distributed: *η *~ *N(0, ω*^*2*^*) *with covariance defined by the inter-individual covariance matrix Ω.

The residual error was described by an additive expression on a log-transformed scale (i.e., proportional error model):

$$\ln(C_{ij})=\ln({\hat{C}}_{ij})+\varepsilon_{ij}$$

where:

*C*_*ij *_is the *j*th measured observation in individual *i*

${\hat{C}}_{ij}$ is the *j*th model predicted value in individual *i*

*ε*_*ij *_is the additive residual random error for individual *i *and measurement *j *and is assumed to be independently and identically distributed

The Bayesian forecasting model utilizes the NONMEM PRIOR subroutine to incorporate population priors into the model. Fixed effects parameters obtained from the final pop PK model were implemented for the initial Bayesian model. Prior distributions of the fixed effects parameters were obtained from the variance-covariance matrix from the final pop PK model as well. Prior distributions for random effects parameters were specified as an inverse Wishart distribution. Clearance was implemented as a mixture model, where a patient is assigned to a population (normal or impaired clearance) based on the probability of that patient belonging to either population given their MTX plasma concentrations. The Bayesian forecasting model was evaluated using MTX plasma concentrations that were not used during model construction. The model reliably predicts future MTX plasma concentrations from two prior concentrations in all patients except a small number who develop renal toxicity at delayed times (> 48 hours). In these patients, the addition of a third concentration after 48 hours increases the precision of the prediction of concentrations at later times.

### Dashboard Design and System Architecture

**Figure 2:**
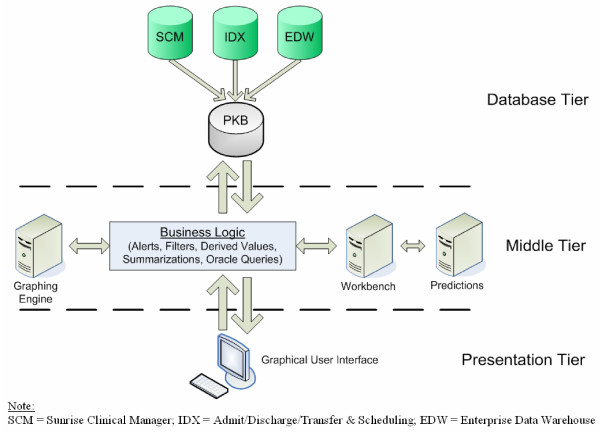
Schematic of three-tier system architecture of hospital pharmacotherapy decision support system comprising a back end database tier, a business logic middle tier and data presentation/user interface front-end tier.

The MTX dashboard was developed based on a three-tier architecture comprising a back end database tier, a business logic middle tier and a data presentation/user interface tier at the front (see Figure 2). The database tier consists of patient records from our electronic medical records system (Sunrise Clinical Manager, SCM) merged with data from patient registration system (IDX), lab data management system (Clarity) and adverse event management system (proprietary). Required data from these systems are extracted and loaded into relational tables within the staging area of the PKB. The data fields are then processed systematically for gaps and manually filled (from patient charts) when any of the missing data are critical for functionality. Such gaps are then noted for future improvements in the data collection process. The level of data validation is minimal and sufficient for testing; a comprehensive data validation approach has been outlined for implementation prior to production release. Various views and summary tables are created from the relational tables for quick retrieval by the application. The current version of the application retrieves data from staging area tables via views and summary tables. Eventually, a multidimensional data mart will permit ad hoc querying and drill down analysis. Upon its completion, data from the staging area will be transformed and loaded into the PKB datamart.

The middle tier consists of rules and processing logic required to collect and prepare data for user presentation (alerts, filters, aggregations, derived values and predictions). Predictions are conducted in an external computational platform – our modeling and simulation (M&S) workbench. This platform can execute code in a variety of languages provided they can run in a batch mode. Of note, the M&S workbench can currently accommodates many of the standard prediction engines used to forecast PK and PK/PD relationships (NONMEM, SAS, SPLUS and R). Details of analytical run processing using NONMEM with the workbench are described below. While the workbench can perform various data processing functions and analytics including generation of plots and figures, it is important to note that all PKB related analytics are gated in the middle tier through logic to ensure that minimally required data sets are available for each patient or sets of patients for meaningful analysis (e.g., appropriate data density to make predictions, etc).

The user interface is a currently web-based and utilizes a combination of HTML, JavaScript and XML content. We are in the process of migrating to an AJAX paradigm and implementing Web 2.0 standards (the new standard for live HTML content). Upon successful transition to Web 2.0, it is possible to readily support more user interface types such as java applet, java application or stay completely within the browser. The Workbench component can co-reside on the same server or installed on another server within the intranet or even hosted anywhere on the internet. The system is designed to transfer data and messages in an authenticated and completely secure manner. When a physician invokes a given forecasting function within PKB, the data required of that patient for the analysis of interest is extracted and written to a flat file in the required application format. The system then edits a previously prepared control file (with job settings and analytical inputs) with necessary changes and makes a call to the Workbench requesting an execution. The communication and data transfer mechanisms are implemented via message queues, thereby ensuring guaranteed delivery, fault tolerance and scalability. The Workbench execution module listening to the queue receives the request and submits the jobs to NONMEM or whatever application has been specified for that analysis by the experts and calls the process manager module to monitor for completion. Upon completion, the process manager calls an application specific parser module to parse and return the results back to the calling application (middle-tier). The middle-tier receives the results and formats it into an XML object and returns the same to the browser for display. If the output of the analysis calls for graphical display of results, the middle-tier sends the unformatted output to the graphing engine and forwards the generated graphs back to the browser for display. The graphs can be setup to support drill downs, mouseovers or invoke further analysis. This ensures that analysis of any kind can be performed via the M&S Workbench to support drug/disease dash boards of varying complexities.

## Results

**Figure 3:**
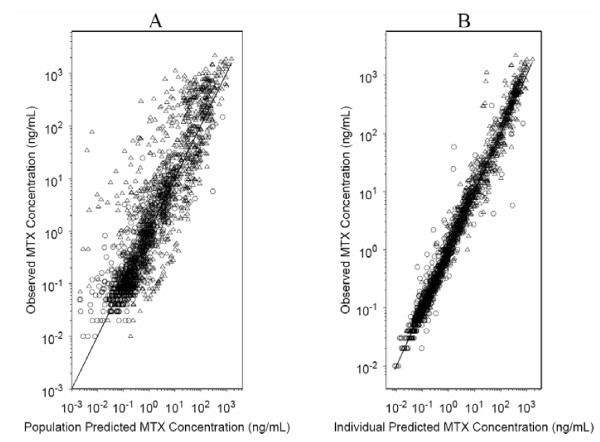
Diagnostic plots from preliminary methotrexate population pharmacokinetic model. (A) Observed versus population predicted concentrations. (B) Observed versus individual predicted concentrations. Open circles represent MTX plasma concentrations from patients predicted to have normal renal function. Open triangles represent patients predicted to have reduced MTX clearance.

The population pharmacokinetic parameters used as initial priors in the initial Bayesian forecasting algorithm that predicts methotrexate drug concentrations are summarized in Table 2. Diagnostic plots from the current population pharmacokinetic model are shown in Figure 3. These plots indicate that the model is adequate to describe the data. Of course the initial parameter set is simply a reference point for the forecasting routine. As patient-specific data (observed MTX concentration-time data relative to an individual dosing event) is added to the data set, the model is executed again as previously described and updated "priors" are generated incorporating the new subject's observations.

**Table 2:** Current population pharmacokinetic parameter priors used to forecast methotrexate plasma concentrations in pediatric patients

**Parameter**	**Units**	**Estimate**	**BSV**
CL_N_	L/h	8.13	41.2%
CL_R_	L/h	2.59	82.0%
V1	L	39.6	21.0%
V2	L	3.94	47.6%
Q	L/h	0.113	7.56%

CL_N _– Clearance in patients with normal renal function

CL_R _– Clearance in patients with reduced renal function

V1 – Volume of distribution in the central compartment

V2 – Volume of distribution in the peripheral compartment

Q – Inter-compartmental clearance

BSV – Between subject variability

**Figure 4:**
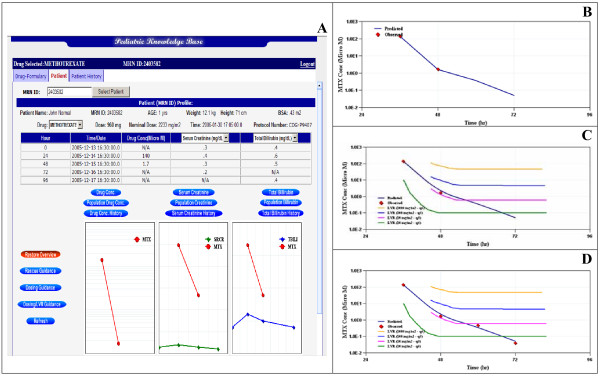
Screen captures from the current MTX dashboard design showing (A) the most recent MTX dose event with the complementary monitored MTX plasma concentrations and safety markers, (B) the MTX exposure projected after the dosing guidance menu button is selected, (C) the view from Figure 4B overlaid against a nomogram used to assess the potential for MTX toxicity with consideration for drug rescue with leucovorin and (D) the update of the model fit when the additional blood collection time points were added to the patient data set.

The prototype methotrexate dashboard module is focused on visualizing the current and recent dosing events relative to markers which indicate the potential for drug-induced toxicity. It also forecasts methotrexate exposures within the current dosing regimen to project the potential for toxicity and provide dosing guidance. The user will access the MTX dashboard from our existing medical records system, Sunrise Clinical Manager (SCM). Eventually, the dashboard will be migrated to the EPIC system which is being integrated with the hospital network in phases. From either system, there will be multiple points of entry with the most likely access coming from the patient medical record number. Figure 4 shows screen captures of the current MTX dashboard design. The dashboard contains three tab views (Drug Formulary, Patient, and Patient History). The details of the entire MTX dashboard will be defined in a separate publication. Briefly, the Drug Formulary tab presents formulary data supplied by Lexicomp restructured in a more web-friendly and content rich layout. The Patient History tab provides historical views of previous experience with an agent in the same patient from previous hospital visits. The first panel (Figure 4A) shows the initial entry screen with the patient tab shown. All modeling and simulation is executed via the patient tab. The initial screen (Figure 4A) shows the most recent MTX dose event with the complementary monitored MTX plasma concentrations and safety markers. Based on input from our pediatric oncology community, the dashboard contains a subset of all data captured within SCM with selections from drop down windows possible. Serum creatinine and total bilirubin concentrations are shown plotted with MTX concentrations from the last dose event. Double clicking on any of the three plots will yield an expanded single plot with axes, scale and units shown.

Figure 4B is the view projected after the dosing guidance menu button is selected. The plot shows the observed 24 and 48 hour MTX plasma concentrations along with the model-predicted MTX exposure (solid line) at these time points and at a subsequent, extrapolated 96 hour point. This extrapolated time point is estimated by calling the MTX population PK model and running the model executable with the patient's previous (observed data) incorporated. The refitted model with updated "patient-specific priors" is then used to project (simulate) the exposure at the 96 hour point, a time when blood collection for MTX plasma concentration determination is normally scheduled in compliance with formulary monitoring practice for MTX.

**Figure 5:**
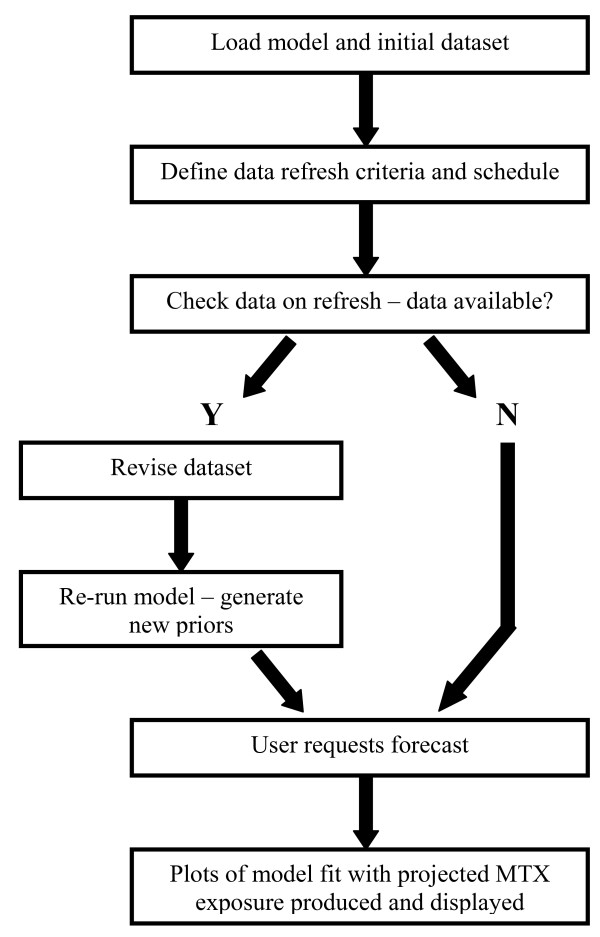
Workflow of MTX dashboard operation.

Figure 4C shows projected view from Figure 4B overlaid against a nomogram used to assess the potential for MTX toxicity with consideration for drug rescue with leucovorin. Several similar nomograms exist for managing MTX drug therapy. Nomograms are aligned to the clinical protocol that the patient is being treated from (protocol is shown in the dashboard in the upper right corner of each screen next to patient demographics. The dashboard contains each of the nomograms used at our institution and so correctly matches the drug exposure views to the nomogram by index to study protocol. Figure 4D illustrates the update of the model fit when the additional blood collection time points were added to the patient data set. The various stages of the MTX dashboard interface including data refresh, model update and output generation are described via the workflow diagram shown in Figure 5. While real-time access is desirable, a scheduled data refresh is more practical. This also removes the burden of data check and model update from the user operation with only calls to produce simulation plots generated by the actual user interface screen.

## Discussion

The development of drug-specific dashboards to educate patient caregivers on principals of clinical pharmacology and guide pharmacotherapy in pediatric populations is likely to yield superior clinical outcomes (fewer medication errors, reduced toxicity, reduced length of hospital stay, etc). While this approach has been advocated for some time and pioneering work by Jelliffe and others[25,26,46] has long demonstrated the clinical benefit of model-based dosing guidance, this research has not yielded any sustainable impact. The dashboard system proposed herein will rely heavily on the integration of modeling and simulation approaches in order to provide meaningful decision analytics to the end user. Our prototype methotrexate dashboard assembles the most clinically relevant patient data into an interface that allows end users to assess relevant biomarkers against drug exposure and forecast future exposures from a given dosing regimen as opposed to waiting to measure such levels via traditional TDM approach. Hence, an earlier assessment of the potential for nephrotoxicity can be made. The underlying population pharmacokinetic model was defined based on limited pediatric data which will be rechallenged prior to final model qualification/validation and production release of the MTX dashboard.

Future considerations for dashboard concepts will include functionality to predict the likelihood of drug interaction with co-administered drugs. By simulating virtual drug interaction studies, we will have the ability to report potential adverse events based on data mining and correlation analysis. We also envision the necessity of expanding the dashboard design construct to accommodate multiple agents considered as treatment options for targeted indications. In this instance, the choice of agent would be a decision criterion to be evaluated prior to the initiation of drug therapy. Our concept here would be to create workflows that considered patient status and previous pharmacotherapy outcomes along with criteria for ranking agent choice depending on the selection attributes. This situation is quite common with antibiotic therapy, which is already receiving attention with respect to commercially available solutions to tracking and prescribing. TheraDoc [47-49], Cereplex [50] and MedMined [51,52] all represent commercial solutions in this arena. TheraDoc mines patient data for trends in infections and suggests courses of action for particular patients, while Cereplex searches for unusual infection patterns and identifies patients requiring changes in therapy and MedMined uses data-mining algorithms to tease out unusual patterns and correlations from patient records and lab tests. The development of decision support systems for managing antibiotic therapy spans several decades now[6,53-55]. Much of the impetus for such systems has been the desire to respond clinically to dynamic changes in local or global bacterial prevalence as well as develop strategies to combat resistance. Likewise, as the landscape of therapeutic options changes with the introduction of new antibiotics, new data on additional indications (e.g., efficacy against new bacterial strains), epidemiologic data on cure rates, global/regional resistance development, and/or the exodus of agents from the market, such systems need some level of continued support beyond information technology. Ownership, governance and preventive maintenance efforts must become formalized for such systems to continue to provide the same level of guidance as when they were first implemented.

The broad array of decision support systems currently employed in hospital settings coupled with those in development highlights the need for robust data integration and flexible decision analytics validated against all possible conditions of use and practice. While the concept of modeling and simulation integration is relatively straightforward, the details of ensuring the performance of these systems, particularly those that impart clinical guidance are complex and require input from IT, clinical pharmacology, pharmacy and clinical practice. The governance of our efforts is overseen by our IRB and therapeutics standards committee but this alone does not ensure the practical issues associated with guiding pharmacotherapy. Given the paucity of information often available to guide pediatric pharmacotherapy, there is a strong desire to "fill-in" such gaps with the best available information available. Likewise, the void in data and knowledge today does not imply that such gaps will remain and decision analytics provided to guide present pharmacotherapy must be revisited as new information becomes available.

## Conclusion

The integration of modeling and simulation algorithms with hospital-based networks to guide the pharmacotherapeutic management of individual patients has great potential to improve outcomes. The benefits of such a system should include improved therapy (efficacy), reduced medication errors, greater appreciation for drug interaction potential, earlier identification of toxicity, and earlier guidance on rescue therapy. Our prototype dashboard concept is part of a broader initiative to develop a pediatric knowledgebase of which dashboards are only one component. At present, dashboards for methotrexate, tacrolimus and vancomycin are at various stages of development. Collaborations with other institutions and investigators should allow the generation of additional dashboards beyond our existing capacity. Such systems, as they are developed, will require a level of support beyond which many hospitals are accustomed as mentioned previously. More importantly, they imply a continued effort from clinical pharmacologists and engineers to implement relevant, new research into these systems to ensure that they continue to perform up to expectations and evolve with advances in drug therapy in pediatrics.

## Competing interests

The author(s) declare that they have no competing interests.

## Authors' contributions

JB provided the pharmacostatistical theoretical framework for modeling and simulation functionality with the decision analytics, is the sponsor for the project on the Chair's Initiative Steering Committee at CHOP and drafted the manuscript. JM created the methotrexate population pharmacokinetic model, provided model qualification and created the forecasting algorithm. MN is the project manager and liaison with the hospital IS team that coordinates interactions with the data managers supporting the electronic medical records systems. KV is the primary programmer responsible for the dashboard interface with the operational data stores, data modeling and Workbench integration. SV is the system engineer on the project responsible for the software and hardware architecture as well as the design requirements and validation effort. All authors read and approved the final manuscript.

## Pre-publication history

The pre-publication history for this paper can be accessed here:


